# A method package for electrophysiological evaluation of reconstructed or regenerated facial nerves in rodents

**DOI:** 10.1016/j.mex.2018.03.007

**Published:** 2018-03-30

**Authors:** Yuichi Takeuchi, Hironobu Osaki, Hajime Matsumine, Yosuke Niimi, Ryo Sasaki, Mariko Miyata

**Affiliations:** a Department of Physiology I (Neurophysiology), Tokyo Women’s Medical University, Tokyo, Japan; b Department of Plastic and Reconstructive Surgery, Tokyo Women’s Medical University, Tokyo, Japan; c Department of Oral and Maxillofacial Surgery, Tokyo Women’s Medical University, Tokyo, Japan

**Keywords:** Compound muscle action potential recordings via reconstructed or regenerated facial nerves, Facial nerve, Reconstruction, Regeneration, Compound muscle action potential, Retrograde tracer, Rat, Mouse, Analysis, Igor Pro, MATLAB, Version control system

## Abstract

Compound muscle action potential (CMAP) recording via reconstructed or regenerated motor axons is a critical examination to evaluate newly developed surgical and regeneration techniques. However, there is currently no documentation on technical aspects of CMAP recordings via reconstructed or regenerated facial nerves. We have studied new techniques of plastic surgery and nerve regeneration using a rat facial nerve defect model for years, standardizing an evaluation pipeline using CMAP recordings. Here we describe our CMAP recording procedure in detail as a package including surgical preparation, data acquisition, analysis and troubleshooting. Each resource is available in public repositories and is maintained as a version control system. In addition, we demonstrate that our analytical pipeline can not only be applied to rats, but also mice. Finally, we show that CMAP recordings can be practically combined with other behavioral and anatomical examinations. For example, retrograde motor neuron labeling provides anatomical evidence for physical routes between the facial motor nucleus and its periphery through reconstructed or regenerated facial nerves, in addition to electrophysiological evidence by CMAP recordings from the same animal.

• *Standardized surgical, recording and analytical procedures for the functional evaluation of reconstructed or regenerated facial nerves of rats, extended to mice.*
• *The functional evaluation can be combined with anatomical evaluations.*
• *The methods described here are maintained in public repositories as version control systems.*

## Graphical abstract

**Figure:**
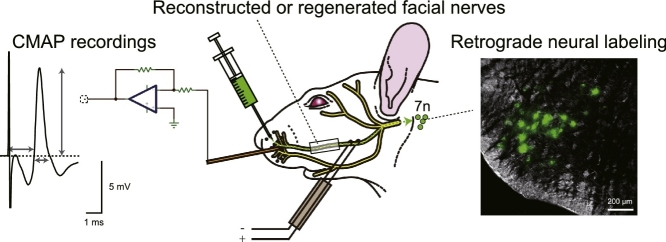

Specifications Table

**Table:** 

Subject area	Medicine and Dentistry
More specific subject area	Reconstruction and regeneration of the facial nerves
Method name	Compound muscle action potential recordings via reconstructed or regenerated facial nerves
Name and reference of original method	Reconstruction or regeneration of the facial nerves of rats.
	* R. Sasaki, S. Aoki, M. Yamato, H. Uchiyama, K. Wada, T. Okano, H. Ogiuchi, Tubulation with dental pulp cells promotes facial nerve regeneration in rats, Tissue Eng. Part A 14(7) (2008) 1141-7.
	* H. Matsumine, R. Sasaki, M. Takeuchi, M. Yamato, H. Sakurai, Surgical procedure for transplanting artificial nerve conduits for peripheral nerve regeneration, Plast. Reconstr. Surg. 128(2) (2011) 95e-97e.
	* H. Matsumine, R. Sasaki, Y. Takeuchi, M. Miyata, M. Yamato, T. Okano, H. Sakurai, Vascularized versus nonvascularized island median nerve grafts in the facial nerve regeneration and functional recovery of rats for facial nerve reconstruction study, J. Reconstr. Microsurg. 30(2) (2014) 127-136.
	* H. Matsumine, Y. Takeuchi, R. Sasaki, T. Kazama, K. Kano, T. Matsumoto, H. Sakurai, M. Miyata, M. Yamato, Adipocyte-derived and dedifferentiated fat cells promoting facial nerve regeneration in a rat model, Plast. Reconstr. Surg. 134(4) (2014) 686-97.
	* Y. Niimi, H. Matsumine, Y. Takeuchi, R. Sasaki, Y. Watanabe, M. Yamato, M. Miyata, H. Sakurai, Effectively axonal-supercharged interpositional jump-graft with an artificial nerve conduit for rat facial nerve paralysis, Plast. Reconstr. Surg. Glob. Open 3 (2015) e416.
Resource availability	CMAPMethods (https://doi.org/10.17632/9g5n35fd3f.1)
	CMAPAnalysis (https://github.com/yuichi-takeuchi/CMAPAnalysis)
	CMAPanalysisMATLAB (https://github.com/hironobu-osaki/CMAPanalysisMATLAB)
	RetrogradeMotorNeuronLabeling (https://doi.org/10.6084/m9.figshare.5445199)

## Method details

### Animals

LEW/Crl rats (200–300 g; RRID:RGD_737932) have been employed in our research on the reconstruction or regeneration of the buccal branch of facial nerves [[1], [2], [3]]. C57BL/6 mice (20–40 g; RRID:IMSR_JAX:000664) were introduced to our research in this study. All experiments conducted were approved by the Animal Care and Use Committee of the Tokyo Women’s Medical University and performed according to the institutional guidelines.

### Surgical procedures for reconstruction or regeneration of the buccal branch of rat facial nerves

Briefly, rats are anesthetized with 4% isoflurane via a nasal mask connected to a Univentor 400 Anesthesia Unit (Univentor, Zejtun, Malta) [4]. A periauricular incision is made on the left side of the face to expose the buccal and marginal mandibular branches of the facial nerves and the parotid gland [1]. The marginal mandibular branch is cut using microsurgical scissors and ligated with 7–0 nylon sutures. After that, the buccal branch is exfoliated from surrounding connective tissues (as shown in Fig. 3D) and a 7 mm-defect is then made on the buccal branch of the facial nerves as previously described [1,4]. This defect could be treated by reconstructing the nerves using an autologous graft from the contralateral buccal branch [2], or by regenerating the nerves using a silicon tube filled with type I collagen solution as a nerve conduit [5]. Further published defect treatments are provided in the Additional information section of the Appendix A., including detailed schematic illustrations and a movie for nerve tube implantations [5,6].

### CMAP recording procedures

**Fig. 1:**
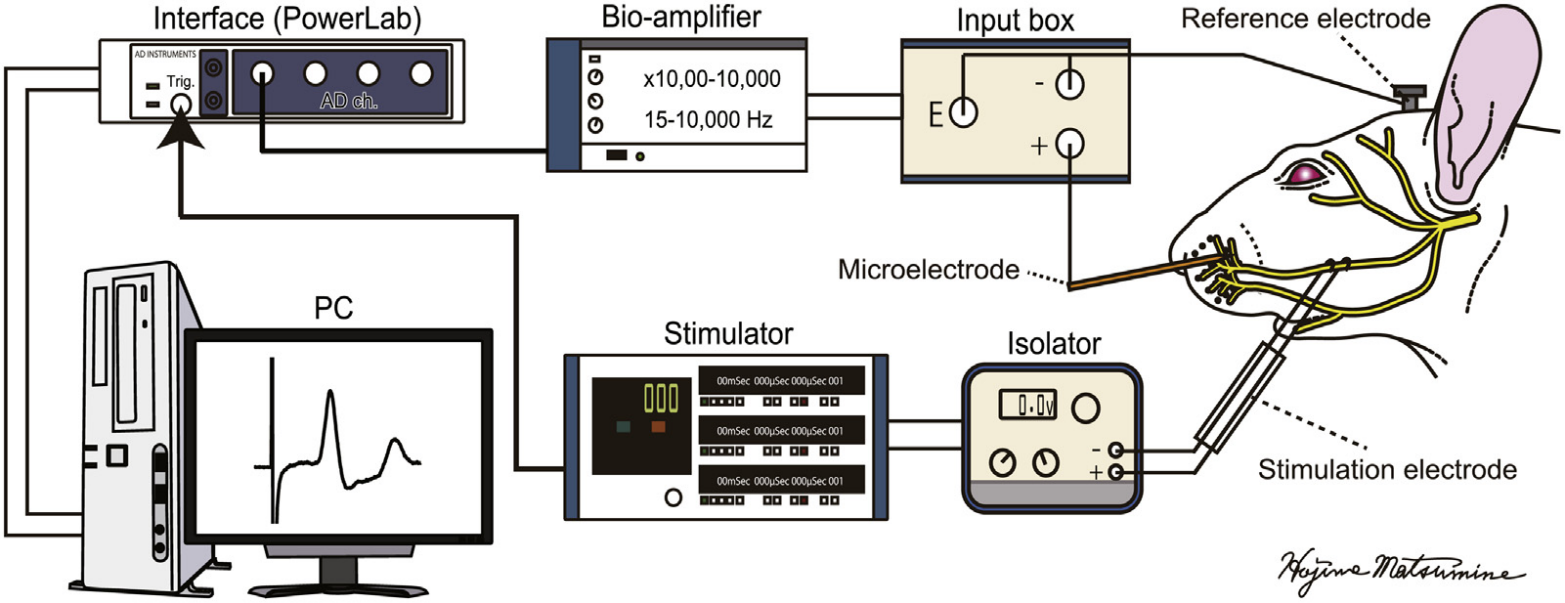
Schematic system overview of a compound muscle action potential (CMAP) recording of vibrissal muscles after stimulation of the buccal branch of facial nerves. A screw-type reference electrode is fixed on the skull and a recording microelectrode is inserted in the whisker pad of an anesthetized rat. Differential potentials between these electrodes are band-pass filtered and amplified with a bio-amplifier and fed to a computer interface. A stimulator delivers TTL signals to trigger data acquisition via the interface. Shortly after beginning the data acquisition process, the stimulator triggers a monopolar square pulse via an isolator to evoke CMAP through the excitation of reconstructed or regenerated facial nerves.

Seven to thirteen weeks after reconstruction or regeneration surgery, the buccal branch can be reconnected with sufficient physical strength to become physiologically functional [1,2,7]. CMAPs from the vibrissal muscles are then able to be recorded after stimulation of the reconstructed or regenerated nerves [8,9]. A schematic overview of these CMAP recordings is shown in Fig. 1. The materials for the recordings are listed in Table 1. Note that those materials on the list could be replaced with comparable ones from other manufacturers (*see* Additional information of the Appendix A.). We provide a hands-on manual for CMAP recordings in the CMAPMethods dataset in Mendeley Data (https://doi.org/10.17632/9g5n35fd3f.1) [10].

**Table 1:** Material list for CMAP recordings. Information on alternatives is provided in the Additional information section of Appendix A.

Name	Type	Company	Catalog Number	Comments
Isoflurane	Reagent	Abbot		Anesthesia
Urethane	Reagent	Wako	050-05821	Ethyl Carbamate
Physiological saline	Reagent	Otsuka Pharma	OTSUKA NORMAL SALINE	
Bio-Amplifier system	Equipment	Nihon Kohden	MEG-6100	Multi-channel system
Amplifier	Equipment	Nihon Kohden	AB-610J	Component
Input box	Equipment	Nihon Kohden	JB-610J	Component
Stimulator	Equipment	Nihon Kohden	SEN-8203	3 ch.
Isolator	Equipment	Nihon Kohden	SS-202J	With SEN-8203
DC temperature controller	Equipment	FHC	40-90-8D	Noise less
Rectal thermistor	Supply	FHC	Mini Rectal Thermistor Probe	With 40-90-8D
Heating pads	Supply	FHC	Heating Pad	With 40-90-8D
Stereotaxic apparatus	Equipment	Narishige	SR-6R	For rats
Stereotaxic apparatus	Equipment	Narishige	SR-6M	For mice
Accessory ear bar	Supply	Narishige	EB-4N	For rats
Accessory ear bar	Supply	Narishige	EB-5N	For mice
Micromanipulator	Equipment	Narishige	MM-3	Holds a recording electrode
Magnet stand	Supply	Narishige	GJ-1	
Iron plate with grand termination	Supply			Put below the stereotaxic apparatus
Operating microscope	Equipment	WILD	Type308795	
Dental drill	Equipment	NSK	Drill Vmax35RV Pack	120 V
Drill bit	Supply	Dentsply	No. 8	
PC	Equipment	Dell	Optiplex910	Windows 10
Interface	Equipment	ADInstruments	PowerLab4/30	USB
Recording software	Software	ADInstruments	LabChart7	
Hook-shaped bipolar stimulus electrodes	Supply	InterMedical	IMM2-220224	Custom made
Stainless steel microelectrode	Supply	FHC	UESMGCSELNNM	9–12 MΩ
Screw-type reference electrode	Supply	Unique Medical	TN204-089B	Custom made
Dental utility wax	Supply	G.C.	UTILITY WAX	
5-0 nylon suture	Supply	Alfresa	EP1105NB45	

#### Booting a typical recording system

**Fig. 2:**
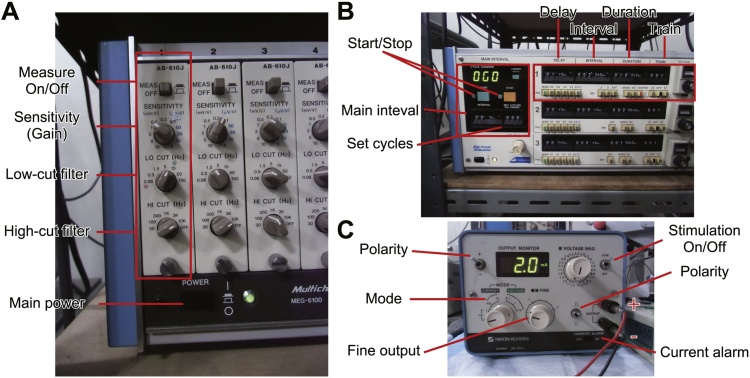
Equipment settings. (A) Multi-channel bio-amplifier. Recommended setting: 1k times amplification, 15–10,000 Hz band-pass filtering. (B) Stimulator. The main interval circuit triggers the first channel at 0.2 Hz, which determines delay (1 ms), interval (not used), duration (100 μs), and the number of stimuli per trigger (single stimulus). (C) Isolator. This circuit optically isolates the stimulation circuit from the other circuits (e.g. stimulator, interface). It also determines the fine amplitude of constant current stimuli with a fine output volume. If the current alarm switch is turned on and the defined current intensity cannot be achieved due to high resistance between the output terminals, there will be a beeping sound.
    Measure: On
    Sensitivity (mV/V): 1–10 (1 mV/V in most cases)
    Low-cut filter (Hz): 15
    High-cut filter (Hz): 10 k
  • Stimulator (Fig. 2B)
    Main interval: 5 s
    Set cycles: 000 (Free run)
    1 Ch. Trig: Main
    Delay: 1 ms
    Interval: 20 ms
    Duration: 100 μs
    Train: 1
    Polarity: +
    Voltage: over 5000 mV
  • Isolator (Fig. 2C)
    Mode: Current 10 mA
    Output monitor: +2.0 mA
    Polarity: +
    Current alarm: On
    Stimulation: On
3 Run data acquisition software (e.g. LabChart, RRID:SCR_001620). Confirm that the gain setting of the software is consistent with the sensitivity (gain) setting of the bio-amplifier (e.g. 1000×) (Fig. 2A). Confirm whether the amplitude of the CMAP waveforms is covered by the voltage range of the analog input channel of the interface (e.g. ±10 V). Other settings can be as follows:
  Sampling: 40 kHz
  Trigger: External
  Duration: 20 ms
  Sweep: ∞

1 Turn on a bio-amplifier and an interface (PowerLab; ADInstruments), and boot a PC (Fig. 1).
2 Confirm that the recording system is set as follows:
  • Bio-amplifier (Fig. 2A)

#### Surgical procedures

**Fig. 3:**
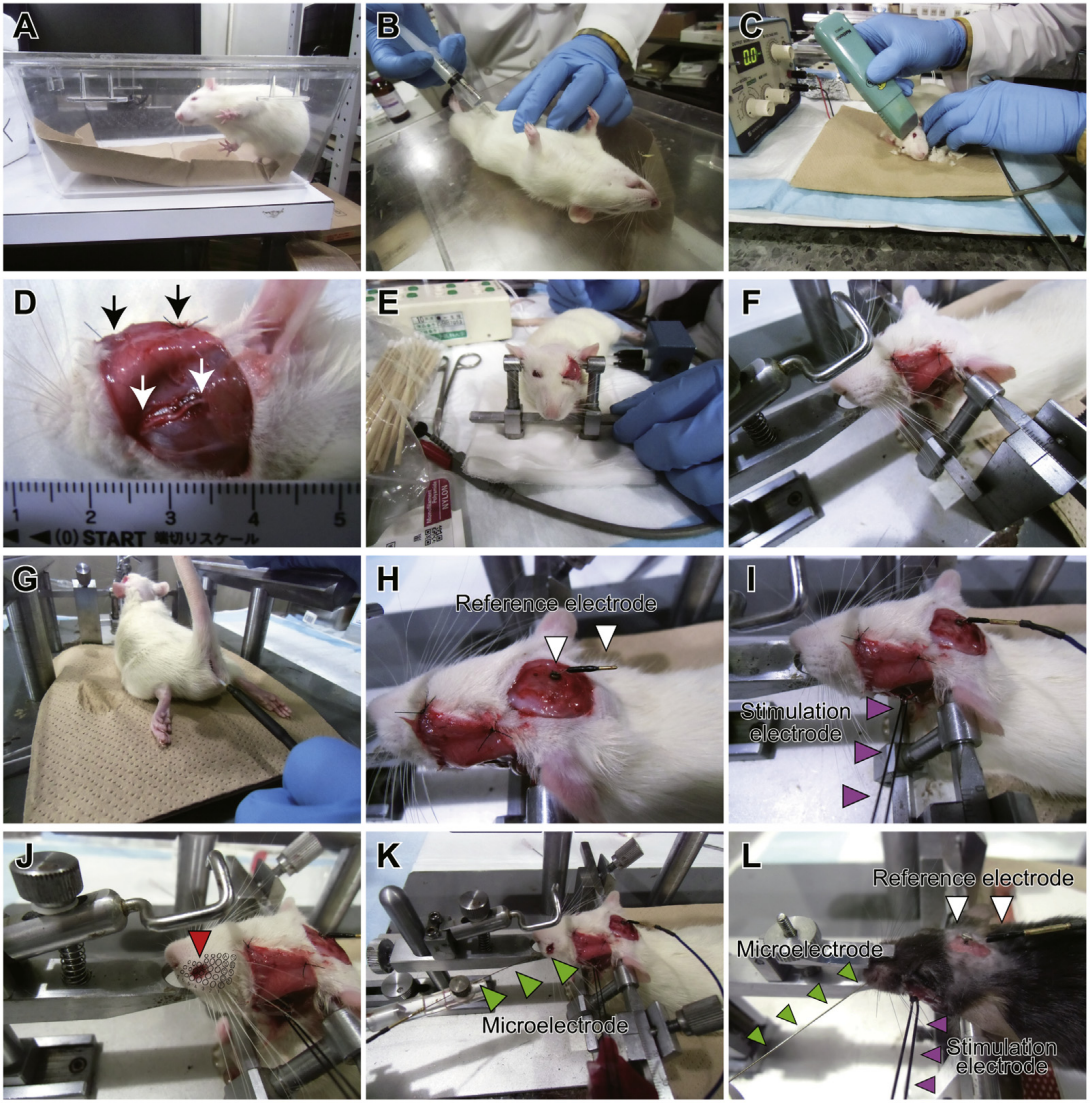
Preparation for CMAP recordings. (A) Introduction of anesthesia with isoflurane. (B) Urethane administration for terminal anesthesia. (C) Shaving. (D) Exposed buccal branch of the facial nerves indicated by white arrows. Black arrows indicate sutures for fixation of the excised skin. (E) Introduction of accessory ear bars. (F) A rat mounted on a stereotaxic apparatus. (G) Introduction of a rectal thermistor for feedback temperature control. (H) A reference electrode fixed on the skull indicated by white arrow heads. (I) A stimulation bipolar electrode hooked to the buccal branch of the facial nerves indicated by magenta arrow heads. Note that anodal and cathodal terminations should be connected to the proximal and distal parts of the nerves, respectively. (J) A small skin incision on the middle of the vibrissal rows C and D of the whisker pad, indicated by a red arrow head. A vibrissal alignment schema is superimposed on the whisker pad. (K) A recording microelectrode inserted into the whisker pad. (L) CMAP recording configuration for mice. The same recording, stimulating and reference electrodes are used. However, the mouthpiece and ear bars are replaced by ones specific to mice.
2 Shave any hairs on the rat’s face and head using a hair clipper, but leave its whiskers intact (Fig. 3C). Whiskers are required to monitor contractions of the vibrissal muscles after electrical stimulations on the nerves.
3 Under an operating microscope, make a preauricular incision on the left side of the face to expose the buccal branch of the facial nerves (Fig. 3D). Incised skin could be stitched using 5–0 nylon sutures to enable a larger field of view. The buccal branch is exfoliated from connective tissues around, resulting in approximately 10 mm-long free nerves.
4 Insert accessory ear bars into the rat’s external auditory canals and put its upper incisors into the mouthpiece of the stereotaxic apparatus (Fig. 3E). Pull the mouthpiece toward the rat’s anterior to stably attach its skull (Fig. 3F).
5 Put a heater mat under the rat and insert the thermistor probe of a DC temperature controller into its rectum (Fig. 3G).
6 Expose the skull on the cerebellum and remove the periosteum on it. Use a dental drill to make a small hole in the skull for a screw-type reference electrode (Fig. 3H). Fix the reference electrode into the skull using a precision driver.
7 Lift the reconstructed or regenerated buccal branch of the facial nerves with a hook-shaped bipolar stimulus electrode (Fig. 3I). Properly position the electrode and then fix it to the stereotaxic apparatus using a dental utility wax. If the reconstructed or regenerated nerve seems very thin and fragile, try not to apply excess tension to it. Put several drops of liquid paraffin or mineral oil on the exfoliated facial nerve to prevent it drying.
8 Connect the stimulation electrode to an isolator (distal to minus and proximal to plus, respectively), and deliver test pulses via a stimulator (Fig. 2B). Adjust the stimulus intensity to 2.0 mA using a digital isolator (Fig. 2C). After that, make sure that the whiskers and vibrissal muscles are moving after each electrical stimulation.
9 Make a skin incision on the middle vibrissal rows C and D of the whisker pad using micro scissors (Fig. 3J) and insert a recording microelectrode into the vibrissal muscles [11]. The microelectrode is then fixed to a micro manipulator (Fig. 3K).
10 Connect the reference and recording microelectrodes to an input box (or pre-amplifier). The reference (–) and ground (earth) terminals are shorted.
11 Start data acquisition (for example, by pressing the ‘START’ button in LabChart software). The software is then waiting for triggers from the stimulator. Launch your stimulator (Fig. 2B) to start the stimulation and recordings.
12 If stable recordings are obtained over ten trials, stop the stimulator and the acquisition software.
13 Save the recordings as a data file in your PC (e.g. with an. adicht extension if you are using LabChart software).
14 Euthanize the rat with an excess amount of isoflurane or transcardially perfuse it for further anatomical examinations.

1 Anesthesia: Put a rat into a transparent box with an acrylic lid and introduce 2–3% isoflurane until the rat does not move including its whiskers (Fig. 3A). After that, urethane is intraperitoneally administered with 1.2 g/kg as a final concentration (Fig. 3B). Separate the dose into several administrations to avoid the unexpected death of the rat.

#### CMAP recordings from mice

Almost the same procedures that were used on rats can be applied to CMAP recordings of mice. To ensure stable physical fixation, use accessory ear bars and a mouthpiece specific to mice (Table 1 and Fig. 3L).

#### Troubleshooting for CMAP recordings

• 50 or 60 Hz line noise: First, check whether your recording system is properly grounded. To avoid ground loop, the GND or chassis terminals of all equipment should be connected and ideally only one of them connected to the earth ideally. However, all equipment may be connected to the earth as the second-best option. Practically, this is much easier and hopefully achieves acceptable noise levels. In many cases of line noise troubles, the reference electrode is not properly fixed on the skull. If this is a problem, make another hole on the skull and fix the reference electrode firmly into the new hole. In addition, unplug the power cables of any ungrounded equipment. A small Faraday cage covering the animal might also help to reduce line noises. If these noises are still unmanageable, an on-line or off-line band elimination filter would be useful (e.g. HumBug; DAGAN).
• 100 or 120 Hz ripple noise: Make sure that your system does not have any ground loops.
• 300–600 Hz pulse-wise noise: It might be the electrocardiogram of your animals. Try not to include their heart between the reference and recording electrodes.
• Continuous DC shift: If you use a DC amplifier, consider an AC differential amplifier instead. Another solution is including a low-cut pre-filter before your DC amplifier.
• The traces after electrical stimulations are out of range and do not come back to the base line immediately: The reference electrode might be loose. Try to make another hole in the skull to secure the reference electrode.
• No physiological response with an intact animal: First, confirm that your amplifier is turned on and a recording microelectrode is connected to a signal terminal of your preamplifier (input box), as shown in Fig. 1. After that, check whether there is a stimulus artifact and if you can see a muscular twitch on the whisker pad at each trial. If stimuli do not seem to be delivered properly and your isolator is power-supplied by batteries, check whether the compliance voltage is high enough to achieve the intended current magnitude.
• No physiological response with a facial nerve-reconstructed or -regenerated animal: In addition to the above-mentioned possibilities, the absence of responses may stem from poor functional recovery after treatments. Check whether there are whisker or whisker pad movements just after each stimulus delivery. If there is still nothing, retract the recording electrode and test another locus on the whisker pad. You might find a small CMAP with a long latency and duration [8].

##### Safety

Always make sure that your recording system is properly grounded. Avoid inflammable anesthetics. For example, diethyl ether.

### Representative results

**Fig. 4:**
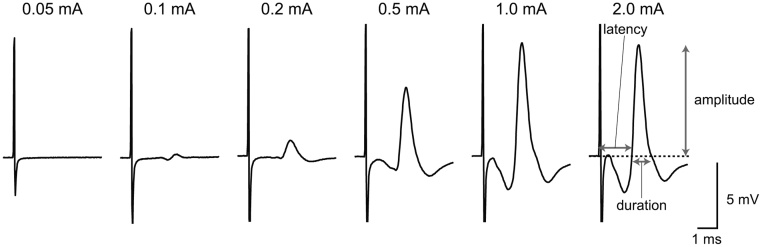
Representative CMAP traces and measurable parameters. Representative CMAP traces recorded from an intact mouse. The numbers above the traces represent stimuli intensities. Note that the amplitude of CMAP is graded until it reaches an upper limit before 1.0 mA. The amplitude is the differential voltage between the baseline (zero) and the positive peak potentials. Latency is the time between the beginning of stimuli artifacts and when the CMAP traces rise over the zero line. Duration is the time between the zero crossing points of the rising and falling trace directions. Recorded potentials relative to the reference electrode is inverted according to the traditional convention.

Representative CMAP recordings from an intact mouse are shown in Fig. 4. Because CMAPs are extracellular field potentials originated from many muscle action potentials, their amplitude is graded with an upper limit in response to increasing stimulus intensity, rather than it being in an all-or-none fashion. In most cases of the buccal branch stimulation of both rats and mice, 2.0 mA is the supramaximal intensity. In addition, because the holistic physiological function of reconstructed or regenerated facial nerves can be evaluated, we always employ 2.0 mA supramaximal stimulation for quantitative evaluations [6,8,9,12,13]. Amplitude, latency and duration of the evoked potentials have been measured as CMAP parameters (Fig. 4). The means ± the standard deviations of these parameters for six intact rats and mice are summarized as Table 2. There were no significant differences between the rats and the mice (*p* > 0.05, two-sample *t*-test; the raw dataset and the entire statistical results are available as supplemental materials).

**Table 2:** Statistical summary of the CMAP parameters of rats and mice.

Group	Amplitude (mV)	Duration (ms)	Latency (ms)
Rat (n = 6)	4.12 ± 2.13	1.17 ± 0.33	1.41 ± 0.37
Mouse (n = 6)	5.02 ± 1.43	0.97 ± 0.17	1.11 ± 0.34

Data were obtained from six intact rats and mice. Values are presented as the mean ± sd. There was no significant difference between rats and mice on each parameter. *P* > 0.05, non-paired *t*-test; two-tailed.

### Analysis

Data saved as LabChart extension (.adicht) can be exported or converted to various formats, including Igor Pro experiments (.pxp) (WaveMetrics, RRID:SCR_000325), MATLAB data files (.mat) (MathWorks, RRID:SCR_001622), Axon binary files (.abf) (Molecular Devices, RRID:SCR_011323), comma-separated value files (.csv) and general ASCII text files. We utilize a custom-made program for Igor Pro: CMAPAnalysis [14], or MATLAB: CMAPanalysisMATLAB [15] for routine analyses.

#### How to analyze CMAP recording data using the CMAP analysis program

**Fig. 5:**
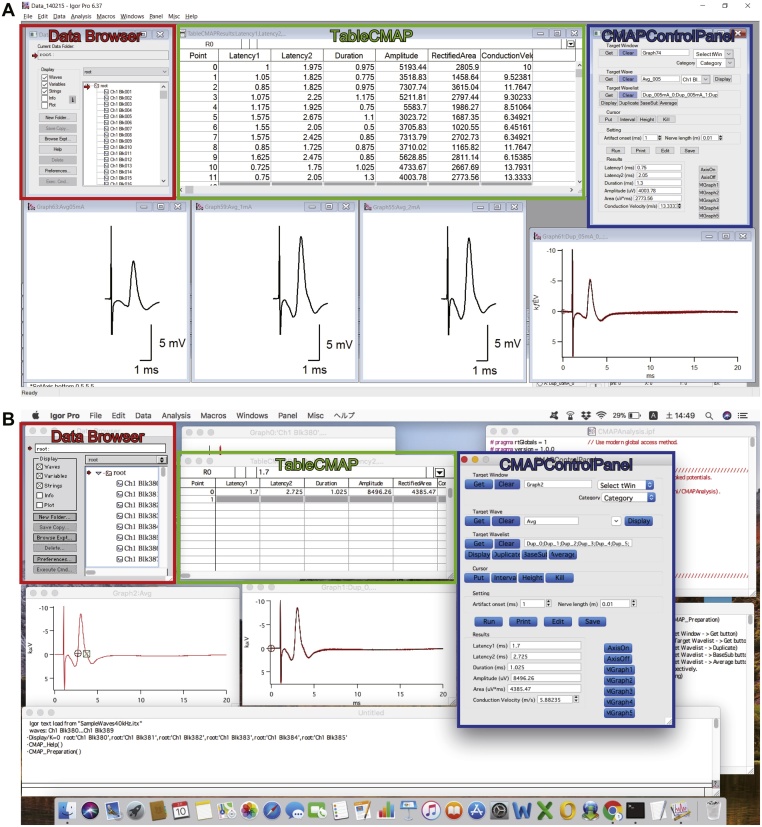
Analysis of CMAP recordings with Igor Pro software. A screenshot of the Igor Pro analytical environment with the CMAPAnalysis add-in program on (A) Windows and (B) Mac OS [14]. CMAPAnalysis offers an integrated control panel (CMAPControlPanel) and TableCMAP, which enables a graphical user interface-based analysis without any programming skills. Results collected on the summary table (TableCMAP) via buttons on the control panel are exported as a csv file and the averaged CMAP traces are automatically built into publication-quality (vectorized) graph windows with scale bars.

CMAPAnalysis is a custom Igor Pro program developed by Y.T. It offers a user-friendly graphical interface to analyze CMAP recordings and runs on both Windows and Mac OS (Fig. 5). The use of CMAPAnalysis does not require programming skills, experimenters only need to do mouse and cursor work. Information on system requirements and installation procedures is available on the GitHub website (https://github.com/yuichi-takeuchi/CMAPAnalysis). Sample raw (Data_140215_Raw.pxp) and analyzed data (Data_140215_Analyzed.pxp) are available in the CMAPMethods dataset (https://doi.org/10.17632/9g5n35fd3f.1) [10]. To analyze your data, follow the steps below.

1 Export your data from LabChart as an Igor Pro experiment (e.g. blahblah.pxp).
2 Open the exported experiment file in Igor Pro.
3 Click ‘CMAP_Preparation’ in the macro menu and the ‘CMAPControlPanel’ window will appear (Fig. 5). Global variables are stored in the root/Packages/CMAP folder in the current Igor Pro experiment.
4 Select multiple waves to be analyzed in ‘Data Browser’ and click ‘Display’ in the short-cut menu after right clicking. Selected waves will be plotted in a new graph window (Fig. 5).
5 Specify the graph as a target window by using the ‘Get’ button in the Target Window group on the control panel (CMAPControlPanel) (Fig. 5).
6 Get a list of source waves by using the ‘Get’ button in the Target Wave group on the control panel.
7 Duplicate the source waves by using the ‘Duplicate’ button in the Target Wavelist group on the control panel.
8 Subtract baseline DC shifts from the source waves by using the ‘BaseSub’ button in the Target Wavelist group on the control panel.
9 Average the duplicated waves by using the ‘Average’ button. The averaged wave will be plotted in a graph window.
10 Place the ‘A’ and ‘B’ cursors on the beginning and ending of the averaged CMAP wave on the graph window, respectively. This should indicate the rising and falling crossings with the zero line (Fig. 4).
11 Set the stimulus artifact onset time (e.g. 1 ms) in the edit box on the control panel.
12 Click the ‘Run’, ‘Print’, and ‘Edit’ buttons on the control panel and you will have summary table results (‘TableCMAP’) and edit boxes on the control panel (Fig. 5). Use the ‘Save’ button on the control panel to export the summary table as a. csv file. A prompt window will appear for this purpose.
13 Prepare publish quality graphs of the averaged traces by using the ‘MGraph1–5’ buttons on the control panel (Fig. 5). The graphs can be exported as. eps files suitable for preparing a figure panel with other vector graphics software (e.g. Adobe Illustrator, RRID:SCR_010279).

##### Troubleshooting for CMAPAnalysis

• Help information is available via the ‘CMAP_Help’ function in the macro menu, or in the ‘CMAPAnalysis’ topic of the ‘Help Browser’ of Igor Pro.
• ‘CMAP_Preparation’ does not appear: The CMAPAnalysis.ipf may not be compiled properly. Follow the installation instructions for CMAPAnalysis (https://github.com/yuichi-takeuchi/CMAPAnalysis) [14].
• ‘CMAPAnalysis’ is not found as a topic in the ‘Help Browser’: The CMAPAnalysis Help.ihf may not be compiled properly. Follow the installation instructions for CMAPAnalysis [14].

#### Statistical analysis

Any statistical packages can be employed for statistical tests. We have utilized commercially available packages including GraphPad Prism (RRID:SCR_002798), Igor Pro and MATLAB. R is widely used for statistical computing software (RRID:SCR_001905) as it is freely available, and it runs on Windows, MacOS and a wide variety of Unix platforms. We have developed a practical R script library for basic biostatistics: RStatisticalTests [16]. In this library, TwoSampleTest.R is used for parametric and non-parametric comparisons between two independent groups. OneWayANOVA.R is used for parametric and non-parametric comparisons between multiple groups, followed by *post-hoc* comparisons.

##### How to use RStatisticalTests for CMAP parameters

System requirements and installation procedures are described on the GitHub website (https://github.com/yuichi-takeuchi/RStatisticalTests). To analyze your data, follow the steps below.

1 The input data file must be a csv file (e.g. AmplitudeData.csv) that has two columns for two independent groups. The first row must include the names of the groups.
2 Make a working directory in your computer (e.g. D:RWD for Windows or/User/<user>/Desktop for Macintosh).
3 To indicate the working directory, edit the first line of a TwoSampleTest.R script file [e.g. AmplitudeTwoSampleTestR.R) as 'setwd("D:RWD")' for Windows, setwd("/User/<user>/Desktop" for Macintosh)], for example.
4 Place the input csv file in the same working directory.
5 Launch the R software.
6 Open the TwoSampleTest.R file (File -> Open Script…).
7 Run the script (Edit -> Run all, or Ctrl + A > Ctrl + R for Windows; Edit > Cmd + A > Cmd + Return for Macintosh).
8 The results will be exported as a text file in the same directory (e.g. AmplitudeResults_TwoSampleTest.txt).
9 Template csv and R files prepared for CMAP recordings (TemplateData.csv.zip and TwoSampleTest.R) are available in the CMAPMethods dataset (https://doi.org/10.17632/9g5n35fd3f.1) [10].

### How to combine CMAP recordings with other examinations

CMAP recordings of facial nerve reconstruction or regeneration can be combined with other behavioral and anatomical examinations. For example, the facial palsy score (obtained by the visual inspection of the symmetry of the vibrissae at rest, the motion of the vibrissae, the symmetry of the nose at rest, and the motion of the nose) [7] can be longitudinally examined during the 7–13 week-long survival time before the CMAP recordings.

During the CMAP recordings, movements of the whiskers can be monitored using a high-speed camera (e.g. 500 Hz frame rate) [17] after each stimulation of the reconstructed or regenerated facial nerves. This provides a correlation between electrophysiological and behavioral observations. Monitoring of whisker movements during CMAP recordings is also useful for detecting a very weak CMAP with a long latency.

After the CMAP recordings, toluidine blue staining and transmission electron microscopy examinations of cross-sections of the reconstructed or regenerated nerves can be conducted to investigate the number of myelinated fibers in the nerve, the diameter of each axon and the thickness of myelin sheath [2,7,12]. Furthermore, retrograde motor neuron labeling via reconstructed or regenerated nerves from the peripheral tissue can be performed to examine whether the reconstructed or regenerated facial nerves are parts of physical route between the facial motor nucleus in the brain stem and its periphery [1,6,9,13,18].

In the following sections, we describe the practical procedures of retrograde tracer experiments along with the CMAP recordings for both rats and mice. Hands-on manuals are available and maintained in the RetrogradeMotorNeuronLabeling dataset (https://doi.org/10.6084/m9.figshare.5445199) [19]. We have employed lipophilic carbocyanine dyes and cholera toxin B subunit (CTB) for rats and mice, respectively [9]. CTB is highly sensitive [20,21] and works with the rat peripheral nervous system as well [22,23]. Furthermore, CTBs labeled with distinct Alexa fluorophores (Thermo Fisher Scientific) could be employed for labeling from different peripheral loci [24]. However, we have not tried CTB for rats so far as CTB and their Alexa conjugates are very expensive; subcutaneous injections of these tracers into rats are also not cost-effective. We have had scientifically sufficient results with carbocyanine dyes for rats [6,9,13,25].

#### For rats

If carbocyanine dyes with different fluorescent spectra are injected separately into different peripheral loci (e.g. DiO, DiI and DiD), motor neurons with different origins are distinctly labeled according to their axonal targets [6,13]. The materials are listed in Table 3. The procedure is outlined below.

**Fig. 6:**
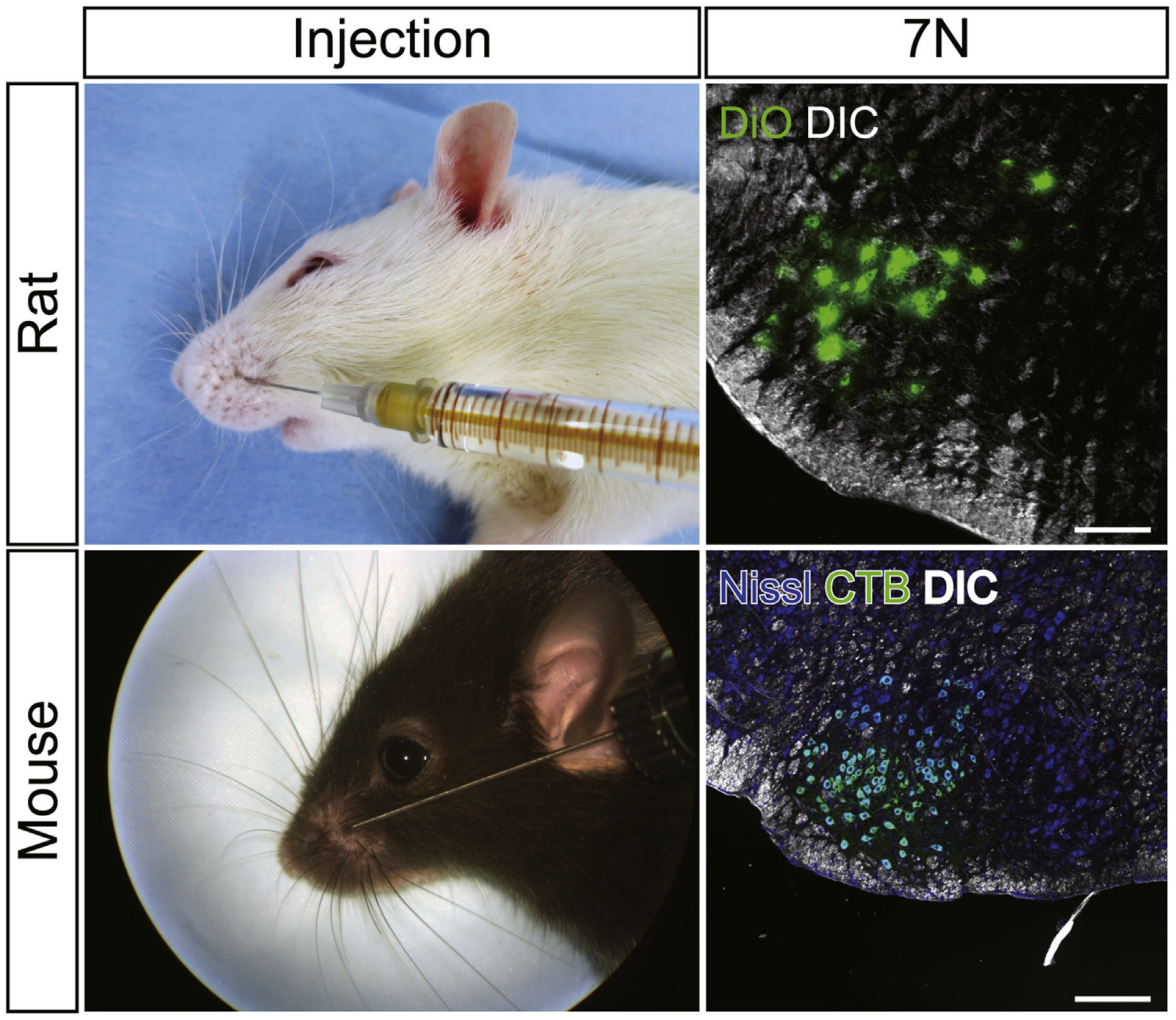
Retrograde motor neuron labeling along CMAP recordings. Lipophilic carbocyanine dyes (e.g. DiO, DiI and DiD) are used to label motor neurons in the facial nerve nucleus from the whisker pad for rats, whereas cholera toxin B subunit is used for mice. Scale bars: 200 μm. Detailed protocols are documented in the text and available in figshare in the RetrogradeMotorNeuronLabeling dataset [19].
4 After a two-week incubation, record the CMAPs as described above.
5 Under deep urethane anesthesia (1.5 g/kg, i.p.), perfuse the rat transcardially with physiological saline followed by 4% paraformaldehyde in 0.1 M PB (pH 7.4). Use the same fixative overnight.
6 Prepare 50 μm-thick coronal brain stem sections with a vibrating blade microtome and harvest them into 12-well culture plates containing 0.1 M PB.
7 Mount the sections on gelatin-coated glass slides and coverslip with an aqueous mounting medium (e.g. PermaFluor^TM^).
8 Observe the sections with an epifluorescence or a confocal microscopy (Fig. 6).
9 Note that brain sections with carbocyanine dyes should not be prepared as frozen sections because freeze-throw cycles injure the plasma membrane of cells and disrupt the integrity of the labeled motor neurons.
10 Avoid mounting media containing glycerol, which can extract membrane-bound dye (e.g. ProLong Gold, VectaShield HardSet^TM^, Aqua-Poly/Mount).
11 Abbreviated solution: PB, phosphate buffer.

1 Dyes are dissolved and stored at 4 °C as a 10% solution in *N,N-*dimethylformamide (DMF).
2 Two weeks before the CMAP recordings, the rats are anesthetized with 4% isoflurane and placed under a dissection microscope.
3 For DiI and DiD, inject 100 μl 1% dye solution into the whisker pads subcutaneously using a Hamilton syringe (Fig. 6). For DiO, inject 200 μl 0.5% solution.

**Table 3:** Material list for retrograde motor neuron labeling with carbocyanine dyes for rats.

Name	Type	Company	Catalog Number	Comments
Isoflurane	Reagent	Abbot		Anesthesia
DiO	Reagent	Thermo Fisher Scientific	D275	Retrograde tracer (Green)
DiI	Reagent	Thermo Fisher Scientific	D282	Retrograde tracer (Red)
DiD	Reagent	Thermo Fisher Scientific	D307	Retrograde tracer (Far red)
DMF	Reagent	Wako	050-05821	Solvent
EtOH	Reagent	Wako	057-00451	Solvent
PermaFluor	Reagent	Thermo Fisher Scientific	TA-030-FM	Aqueous mounting medium
25 μl Hamilton syringe	Supply	Hamilton	80401	Luer Tip
Vibratome	Equipment	Leica Microsystems	VT1000S	Vibrating blade microtome

#### For mice

We have developed highly sensitive retrograde labeling of facial motor neurons for mice along with CMAP recordings. This method not only labels motor neurons, but also sensory afferent fibers to the trigeminal nuclei [21]. Materials are listed in Table 4.

**Table 4:** Material list for retrograde motor neuron labeling with cholera toxin B subunit for mice.

Name	Type	Company	Catalog Number	Comments
Ketamine	Reagent	Daiichi-Sankyo	KETALAR	Anesthesia: Strictly regulated.
Xylazine	Reagent	Sigma-Aldrich	X1251	Sedating drug
Cholera Toxin B subunit	Reagent	Sigma-Aldrich	C9905	Retrograde tracer. Need documentation to purchase.
Microneedle syringe	Supply	WPI	NANOFIL	Comes with 26 gauge needle.
Cryo-blocking medium	Supply	Sakura Finetek	Tissue-Tek^TM^ O.C.T. compound^TM^	Transparent
Cryomold	Supply	Sakura Finetek	Tissue-Tek^TM^ Cryomold^TM^	10 mm × 10 mm × 5 mm
Blocking serum	Reagent	Vector Lab	S-5000	Normal rabbit serum
1^st^ antibody	Reagent	List Biological Lab	703	Go anti-CTB
				RRID:AB_10013220
2^nd^ antibody	Reagent	Vector Lab	BA-5000	Rb anti-Go IgG, biotinylated
Streptavidin	Reagent	Thermo Fisher Scientific	S21375	Alexa Fluor 633-conjugated
Fluoro Nissl solution	Reagent	Thermo Fisher Scientific	N-21470	Blue
Cryostat	Equipment	Leica Microsystems	CM1860	Freezing microtome

##### Injection

1 Two to four days before the CMAP recordings, the mice are anesthetized with a ketamine/xylazine cocktail (80/10 mg/kg, i.p) and placed under a dissecting microscope.
2 Fill a microneedle syringe (NANOFIL; WPI) with 1% CTB solution in 50 mM PBS and inject 5 μl of the solution into the whisker pads subcutaneously (Fig. 6).
3 After two to four days of incubation, record the CMAPs as described above.

##### Perfusion, cryo-embedding, sectioning

1 Under deep pentobarbital anesthesia (120 mg/kg, i.p.), perfuse the mice transcardially with ice-cold saline, followed by 4% paraformaldehyde and 0.2% picric acid in 0.1 M PB (pH 7.2–7.3). Use the same fixative overnight.
2 Infiltrate the brain with sucrose gradient (10–30%).
3 Cryo-embed the brain into a cryomold filled with O.C.T. compound on −80 °C acetone solution with dry ice.
4 Prepare 20-μm-thick coronal brain stem sections using a freezing microtome and harvest them into 24-well culture plates containing an anti-freeze solution (30% glycerol, 30% ethylene glycol, 40% PBS).
5 PBS wash.

##### Staining in free-floating configuration (room temperature, light shielded)

1 10% NRS in PBS-XCR, 30 min on shaker.
2 1:10,000 dil. Go anti-CTB (703, List) in PBS- XCR, overnight on shaker.
3 PBS-X wash (quick × 1, 10 min × 2).
4 1:400 dil. biotinylated Rb anti-Go IgG (Vector) in PBS-XCR, 2 h on shaker.
5 PBS-X wash (quick × 1, 10 min × 2).
6 1:400 dil. Alexa 633-conjugated streptavidin in PBS-X, 2 h on shaker
7 PBS wash (quick × 1, 10 min × 2).

##### Counterstaining with fluorescent Nissl

• 1:150 dil. fluorescent Nissl solution (blue), 40–60 min.
• PBS wash (quick × 1, 10 min × 2).

##### Mounting, cover-slipping, observation

• Mount the sections on gelatin-coated glass slides and air-dry for 30 min.
• Coverslip with 50% (v/v) glycerol and 2.5% (w/v) DABCO (1,4-diazabicyclo [2.2.2] octane) in PBS.
• Observe the sections with an epifluorescence or a confocal microscopy (Fig. 6).
• Abbreviated solutions: PB, phosphate buffer; PBS, 0.1 M phosphate buffered saline; PBS-X, 0.3% Triton-X 100 in PBS; PBS-XCR, 0.12% λ-carrageenan, 1% normal rabbit serum, 0.02% sodium azide in PBS-X.

## Conflicts of interest

The authors declare no conflicts of interest.
