# Supplementary material for: A method package for electrophysiological evaluation of reconstructed or regenerated facial nerves in rodents
Source: MethodsX. 2018 Mar 30;5:283–98. doi: 10.1016/j.mex.2018.03.007 (PMC6055010; doi:10.1016/j.mex.2018.03.007)
Supplement: Supplementary file 3 [file mmc3.docx]

# Supplementary data

1. CMAPRecDataset.csv: The raw dataset values of CMAP amplitudes, duration and latency of six intact rats and mice.

2. StatisiticalResults.txt: The statistical output results on the raw measured values comparing rats and mice.

# Additional information

## Research in the reconstruction and regeneration of the defective buccal branch of the facial nerves in rats

Reconstruction using vascularized nerve graft: Matsumine, H. et al. 2014 [1].

Reconstruction using end-to-side loop graft: Matsumine, H. et al. 2014 [2].

Regeneration with dental pulp cells: Sasaki et al. 2008, 2011, 2014 [3-5].

Regeneration with dedifferentiated fat cells: Matsumine, H. et al. 2014 [6].

Regeneration with adipose-derived stem cells: Watanabe, Y. et al. 2014 [7].

Regeneration with axonal supercharge: Niimi, Y. et al. 2015, 2018 [8, 9].

## Interpretations of CMAP results:

The triphasic response shown in Figure 4 is a typical CMAP wave form. The reason for this is as follows: the microelectrode placed in the vicinity of vibrissal muscles derives dynamic local field potentials of extracellular spaces around the excitable muscles, as the extracellular medium acts as a volume conductor [10]. When the buccal branch of the facial nerves is stimulated and excitatory postsynaptic potentials are large enough, action potentials occur across plasma membranes around neuromuscular junctions of many vibrissal muscle fibers. They start spreading on the membranes as a consequence. The excitation wavefronts of spreading action potentials (at which fast inward transmembrane currents occur) give rise to strong electrical dipoles which cause a significant extracellular field. First, when the excitation wavefronts are approaching the recording electrode, the recording site acts as a source of the dipoles and gets relatively positive to the reference electrode on the skull (the first downward deflection of the representative traces in Figure 4). Second, the excitation wavefronts arrive at the recording site and it becomes a sink and get negative (the upward deflection in Figure 4). After that, the recording site acts as a source of leaving excitation wavefronts and gets positive to the reference again (the second downward deflection).

The amplitude of CMAP is supposed to represent the numbers of muscle fibers firing below a recording site, which directly correlates with the magnitude of local field potentials. Latency includes both the action potential conduction time along reconstructed or regenerated facial nerves and the synaptic delay at neuromuscular junctions. Generally, both reconstructed and regenerated facial nerves have thinner myelin sheaths and smaller axon diameters compared with those of intact or autografted nerves [1, 5, 8]. Thinner myelin sheaths and smaller axon diameters lead to increased membrane capacitances and intracellular cytoplasmic resistance along each axon, respectively. Because membrane capacitance and axonal length resistance are inversely proportional to conduction velocity of action potentials, the morphological changes observed in reconstructed and regenerated facial nerves would be the causes of the increased latency.

The duration of CMAP may reflect the magnitude of the synchronization of action potentials from many muscle fibers. It could also be affected by current densities through voltage-dependent sodium channels and voltage-dependent potassium channels, which are respectively responsible for the rising and falling phases of muscle action potentials. Because we did not observe duration changes in most cases, this parameter seems quite robust (however, see also Matsumine, H. et al. 2014 [1]).

## Supply, equipment and software used for CMAP recordings

### Supply

*Microelectrodes*

As recording microelectrodes, low-impedance (approx. 1–15 MΩ) metal electrodes are optimal for CMAP recordings. Typical microelectrode material is stainless steel or tungsten. In addition to the FHC UE series shown in Table 1, several other commercial products are:

- Alpha Omega, acute electrodes (<https://alphaomega-eng.com/electrodes-arrays/acute-electrode.html>).
- A-M Systems, epoxy or Parylene C-insulated single microelectrodes (<http://www.a-msystems.com/s-14-electrodes.aspx>).
- Microprobes, single electrode (<https://www.microprobes.com/products/metal-microelectrodes/single-electrodes>).

*Reference electrodes*

Screw-type reference electrodes can be made from stainless steel machine screws and thin multi-strand wires.

- Antrin Miniature Specialties Inc, 000-120×1/16-machine screws (<http://www.antrinonline.com/products2.htm>).
- Intermedical, IMS-124, (<http://www.intermedical.co.jp/index_eng.html>).
- Phoenix Wire Inc, AWG 36- Triple PTFE-coated wire. (<https://www.phoenixwireinc.com/ptfe-coated-wire.html>).

*Note that soldering on the head of stainless steel machine screws needs pre-treatment with inorganic acid flux (e.g. Baker’s Soldering Fluid No. 3).

### Equipment

*Low-cost bio-amplifiers*

- High-sensitivity amplifier (MEG-5100; Nihon Kohden).
- Differential extracellular amplifier (EX1; DAGAN).
- AC/DC differential amplifier (3000; A-M Systems).
- Differential amplifier (DP-301; Warner).
- AC differential amplifier (MDA-2; Bak Electronics).

*Analog filters*

- Multifunction filter (3611; NF Corporation).
- Fixed frequency filter (DV-04; NF Corporation).
- Notch filter (Hum Bug; DAGAN).
- Notch filter (EX-Notch; DAGAN).

*Passive and active analog filters are easily constructed with very cheap electrical components [11, 12].

*Stimulators and isolators*

Stimulators are not indispensable if triggers for isolators are software-controlled.

- One-channel stimulator (S-900; DAGAN).
- Eight-channel stimulator (Master-8; AMPI).
- One-channel stimulator and isolator (SEN-5201; Nihon Kohden).
- Two-channel stimulator and isolator (STG-4002; Multichannel Systems).
- Digital stimulus isolator (SS-203J; Nihon Kohden).
- Digital stimulus isolator (ISO-Flex; AMPI).
- Digital stimulus isolator (BSI-2A; Bak Electronics).
- Analog stimulus isolator (S-910; DAGAN).
- Analog stimulus isolator (BSI-1A; Bak Electronics).
- Analog stimulus isolator (2200; A-M Systems).

*Computer interfaces and data acquisition software*

- PowerLab with LabChart software (ADInstruments).
- DigiData with pCLAMP software (Molecular Devices).
- InstruTECH interface and PATCHMASTER software etc. (HEKA Elektronik; RRID:SCR_000034).
- Micro 1401-3 with Spike2 software (CED; RRID:SCR_000903).
- NI DAQ board and LabView software (National Instruments; RRID:SCR_014325).

*General data acquisition software*

- MATLAB and Data Acquisition Toolbox work with NI DAQ interfaces (MathWorks). We provide a general-purpose MATLAB GUI which helps data acquisition using NI DAQ interfaces: NiDaqControlPanel (<https://github.com/yuichi-takeuchi/NiDaqControlPanel>) [13].
- Igor NI DAQ Tools MX works with NI DAQ interfaces (WaveMetrics).
- Igor Pro XOPs (which are freely available from HEKA) offer data acquisition capabilities for Igor Pro software with InstruTECH interfaces, including LIH8+8, ITC18, ITC16 (<http://www.heka.com/downloads/downloads_main.html#down_xops>). We provide versatile Igor Pro GUIs using the XOPs with ITC18 and ITC16: tClamp18 (<https://github.com/yuichi-takeuchi/tClamp18>) [14] and tClamp16 (<https://github.com/yuichi-takeuchi/tClamp16>) [15], respectively.
- AxoGraph works with DigiData, InstruTECH, and NI DAQ interfaces (<https://axograph.com/>).

# Supplementary references

[1] H. Matsumine, R. Sasaki, Y. Takeuchi, M. Miyata, M. Yamato, T. Okano, H. Sakurai, Vascularized versus nonvascularized island median nerve grafts in the facial nerve regeneration and functional recovery of rats for facial nerve reconstruction study, J Reconstr Microsurg 30(2) (2014) 127-136.

[2] H. Matsumine, R. Sasaki, Y. Takeuchi, Y. Watanabe, Y. Niimi, H. Sakurai, M. Miyata, M. Yamato, Unilateral multiple facial nerve branch reconstruction using “end-to-side loop graft” supercharged by hypoglossal nerve, Plast Reconstr Surg Glob Open 2 (2014) e240.

[3] R. Sasaki, S. Aoki, M. Yamato, H. Uchiyama, K. Wada, T. Okano, H. Ogiuchi, Tubulation with dental pulp cells promotes facial nerve regeneration in rats, Tissue Eng Part A 14(7) (2008) 1141-1147.

[4] R. Sasaki, S. Aoki, M. Yamato, H. Uchiyama, K. Wada, H. Ogiuchi, T. Okano, T. Ando, PLGA artificial nerve conduits with dental pulp cells promote facial nerve regeneration, J Tissue Eng Regen Med 5(10) (2011) 823-830.

[5] R. Sasaki, H. Matsumine, Y. Watanabe, Y. Takeuchi, M. Yamato, T. Okano, M. Miyata, T. Ando, Electrophysiologic and functional evaluations of regenerated facial nerve defects with a tube containing dental pulp cells in rats, Plast Reconstr Surg 134(5) (2014) 970-978.

[6] H. Matsumine, Y. Takeuchi, R. Sasaki, T. Kazama, K. Kano, T. Matsumoto, H. Sakurai, M. Miyata, M. Yamato, Adipocyte-derived and dedifferentiated fat cells promoting facial nerve regeneration in a rat model, Plast Reconstr Surg 134(4) (2014) 686-697.

[7] Y. Watanabe, R. Sasaki, H. Matsumine, M. Yamato, T. Okano, Undifferentiated and differentiated adipose-derived stem cells improve nerve regeneration in a rat model of facial nerve defect, J Tissue Eng Regen Med 11(2) (2014) 362-374.

[8] Y. Niimi, H. Matsumine, Y. Takeuchi, R. Sasaki, Y. Watanabe, M. Yamato, M. Miyata, H. Sakurai, Effectively axonal-supercharged interpositional jump-graft with an artificial nerve conduit for rat facial nerve paralysis, Plast Reconstr Surg Glob Open 3 (2015) e416.

[9] Y. Niimi, H. Matsumine, Y. Takeuchi, H. Osaki, S. Tsunoda, M. Miyata, M. Yamato, H. Sakurai, A collagen‐coated PGA conduit for interpositional‐jump graft with end‐to‐side neurorrhaphy for treating facial nerve paralysis in rat, Microsurgery (2018) Epub ahead of print.

[10] G. Buzsáki, C.A. Anastassiou, C. Koch, The origin of extracellular fields and currents--EEG, ECoG, LFP and spikes, Nat Rev Neurosci 13(6) (2012) 407-20.

[11] P. Scherz, S. Monk, Practical Electronics for Inventors, 4th ed., McGraw-Hill Education TAB, New York, 2016.

[12] Y. Takeuchi, ActiveBandPassFilter, figshare, v1.0.0, 2017. https://doi.org/10.6084/m9.figshare.5455702

[13] Y. Takeuchi, NiDaqControlPanel, Zenodo, v1.1.0, 2017. http://doi.org/10.5281/zenodo.831843

[14] Y. Takeuchi, tClamp18, Zenodo, v0.1.0-alpha, 2017. http://doi.org/10.5281/zenodo.805897

[15] Y. Takeuchi, tClamp16, Zenodo, v0.0.0-alpha, 2017. http://doi.org/10.5281/zenodo.805901
